# RanBP2 Modulates Cox11 and Hexokinase I Activities and Haploinsufficiency of *RanBP2* Causes Deficits in Glucose Metabolism

**DOI:** 10.1371/journal.pgen.0020177

**Published:** 2006-10-27

**Authors:** Azamat Aslanukov, Reshma Bhowmick, Mallikarjuna Guruju, John Oswald, Dorit Raz, Ronald A Bush, Paul A Sieving, Xinrong Lu, Cheryl B Bock, Paulo A Ferreira

**Affiliations:** 1 Third Wave Technologies, Madison, Wisconsin, United States of America; 2 Department of Anatomy, Cell Biology, and Neurobiology, Medical College of Wisconsin, Milwaukee, United States of America; 3 Department of Ophthalmology, Duke University Medical Center, Durham, North Carolina, United States of America; 4 National Institutes of Health, National Institute on Deafness and Other Communication Disorders/National Eye Institute, Bethesda, Maryland, United States of America; 5 National Institutes of Health, National Eye Institute, Bethesda, Maryland, United States of America; 6 Duke Comprehensive Cancer Center, Duke University Medical Center, Durham, North Carolina, United States of America; 7 Department of Molecular Genetics and Microbiology, Duke University Medical Center, Durham, North Carolina, United States of America; Johns Hopkins University School of Medicine, United States of America

## Abstract

The Ran-binding protein 2 (RanBP2) is a large multimodular and pleiotropic protein. Several molecular partners with distinct functions interacting specifically with selective modules of RanBP2 have been identified. Yet, the significance of these interactions with RanBP2 and the genetic and physiological role(s) of RanBP2 in a whole-animal model remain elusive. Here, we report the identification of two novel partners of RanBP2 and a novel physiological role of RanBP2 in a mouse model. RanBP2 associates in vitro and in vivo and colocalizes with the mitochondrial metallochaperone, Cox11, and the pacemaker of glycolysis, hexokinase type I (HKI) via its leucine-rich domain. The leucine-rich domain of RanBP2 also exhibits strong chaperone activity toward intermediate and mature folding species of Cox11 supporting a chaperone role of RanBP2 in the cytosol during Cox11 biogenesis. Cox11 partially colocalizes with HKI, thus supporting additional and distinct roles in cell function. Cox11 is a strong inhibitor of HKI, and RanBP2 suppresses the inhibitory activity of Cox11 over HKI. To probe the physiological role of RanBP2 and its role in HKI function, a mouse model harboring a genetically disrupted *RanBP2* locus was generated. *RanBP2^−/−^* are embryonically lethal, and haploinsufficiency of *RanBP2* in an inbred strain causes a pronounced decrease of HKI and ATP levels selectively in the central nervous system. Inbred *RanBP2^+/−^* mice also exhibit deficits in growth rates and glucose catabolism without impairment of glucose uptake and gluconeogenesis. These phenotypes are accompanied by a decrease in the electrophysiological responses of photosensory and postreceptoral neurons. Hence, RanBP2 and its partners emerge as critical modulators of neuronal HKI, glucose catabolism, energy homeostasis, and targets for metabolic, aging disorders and allied neuropathies.

## Synopsis

The Ran-binding protein 2 (RanBP2) is a large protein with several domains. Although several protein partners were found to interact with selective domains of RanBP2, none to this date were found toward its large leucine-rich domain (LD). Cell-based experiments support several roles of RanBP2 in cell function, such as the production of functional proteins, control of protein trafficking between the nuclear and cytosol compartments, and control of multiple facets underlying cell division. Still, the genetic and physiological implications of the interactions between RanBP2 and its partners and of the function of RanBP2 in a whole-animal model remain elusive. The authors report the identification of two novel mitochondrial partners of the LD of RanBP2, Cox11 and hexokinase type I (HKI); and with multidisciplinary approaches probe the role of RanBP2 and its LD on Cox11, HKI, and functions allied to these. The authors found that RanBP2 exhibits chaperone activity toward HKI and Cox11. RanBP2 and Cox11 profoundly modulate HKI activity. Moreover, partial loss-of-function of *RanBP2* in a mouse model induces deficits in growth rates and breakdown of glucose, promotes the down-regulation of HKI and ATP levels selectively in the central nervous system, and impairs visual function. These findings support a critical role of RanBP2 and its partners in metabolic processes and allied disease states.

## Introduction

The RanBP2/Nup358 is a unique vertebrate and large scaffold protein comprised of multiple structural and functional domains [1–4]. Several roles of RanBP2 have emerged that implicate RanBP2 in nucleocytoplasmic trafficking [3,5], protein biogenesis [6,7], the formation of the mitotic spindle, assembly of the nuclear envelope [8], and the integration of the nuclear envelope breakdown with kinetochore formation and maturation during early mitotic progression [9]. The specific interaction of RanBP2 with a diverse set of partners likely reflects a pleiotropic role of RanBP2 in cell function, possibly through the integration of multiple pathways. On the other hand, the cell (tissue)-selective interaction of RanBP2 with some of its partners may also impart cell-restricted roles to RanBP2. For example, the Ran-binding domains RBD_n=1-4_ of RanBP2 associate with the nuclear import co-receptor, importin-β [10,11], and antibodies against RanBP2 inhibit the nuclear import pathway in HeLa cells [3]; but such a role seems dispensable in *Xenopus* oocytes [12]. In addition, the combination of the C-terminal domains, RBD4 and CY (of RanBP2), associates with a subset of G protein-coupled receptors, the red/green opsin, expressed in photosensory neurons and enhances opsin functional production [6,7], while the interaction of the KBD of RanBP2 with a subset of the conventional microtubule-based motor proteins, the kinesins, KIF5B and KIF5C, occurs selectively in the central nervous system (CNS) [13].

A diverse set of additional molecular partners, each associating specifically with a selective domain of RanBP2, are likely to impart and integrate additional roles to RanBP2. For example, the cyclophilin-like domain (CLD), the internal repeat (W1W2/IR), and zinc-finger rich (ZnF) domains of RanBP2 associate specifically with components of the 19S cap of the proteasome [14], the E2 SUMO-1-conjugating enzyme (Ubc9) [15], and the nuclear export receptor, CRM1/exportin-1 [16], respectively. RanBP2 itself was also found to exhibit SUMO1 E3 ligase activity [17], supporting a direct link between RanBP2-mediated SUMO-1 substrate modification and relocation of SUMO-1 modified cytosolic substrates (e.g., RanGAP) to the cytosolic face of the nuclear pore complex (NPC) [18]. Finally, RanBP2 was also found to localize prominently to the mitochondria-rich ellipsoid subcellular compartment of photosensory neurons [19] and to RanGTPase-restricted foci along cytoplasmic tracks [19], in addition to its localization at cytoplasmic fibrils emanating from nuclear pores [2,3,10,12,19].

Emerging evidence supports that the CNS-selective effects of RanBP2 may also underlie the pathogenesis of certain neuropathies. Parkin is ubiquitously expressed and interacts with the Ubc9-interacting domain of RanBP2 [20]. This promotes the ubiquitination and degradation of RanBP2 [20], possibly via the interaction of the CLD domain of RanBP2 with the 19S cap subunits of the proteasome [14]. Interestingly, the small yeast Ran-binding protein 1 (RanBP1), Yrb1p, with strong homology to the Ran-binding domains of RanBP2, is also required for cell-cycle regulated protein degradation [21]. Parkin, like RanBP2 [17], has E3-ligase activity [22–24]; and loss-of-function of parkin leads to early onset autosomal recessive juvenile Parkinsonism [25]. While the dopaminergic neuronal-restricted effects of mutations in parkin are not understood [25], *parkin^−/−^* mice exhibit mitochondrial dysfunction and energy and growth deficits, and a fraction of parkin localizes to the cytoplasmic face of the mitochondria, where together with other partners is thought to promote the degradation of selective mitochondrial substrates and to exert a neuroprotective function [26–29]. These data suggest that deficits in RanBP2 and parkin may share (patho) physiological pathways and support a multifaceted role of RanBP2. Yet clear functions of RanBP2 in animal and cell physiology remain elusive.

Here, we report on the identification and function of two novel partners of RanBP2, Cox11 and hexokinase type I (HKI), which interact with a large and orphan domain of RanBP2, the leucine-rich domain (LD); and on the outcome of partial loss-of-function of RanBP2 on HKI, animal physiology, and glucose/energy metabolism.

## Results

### The LD of RanBP2 Interacts with Cox11 and HKI

**Figure 1:**
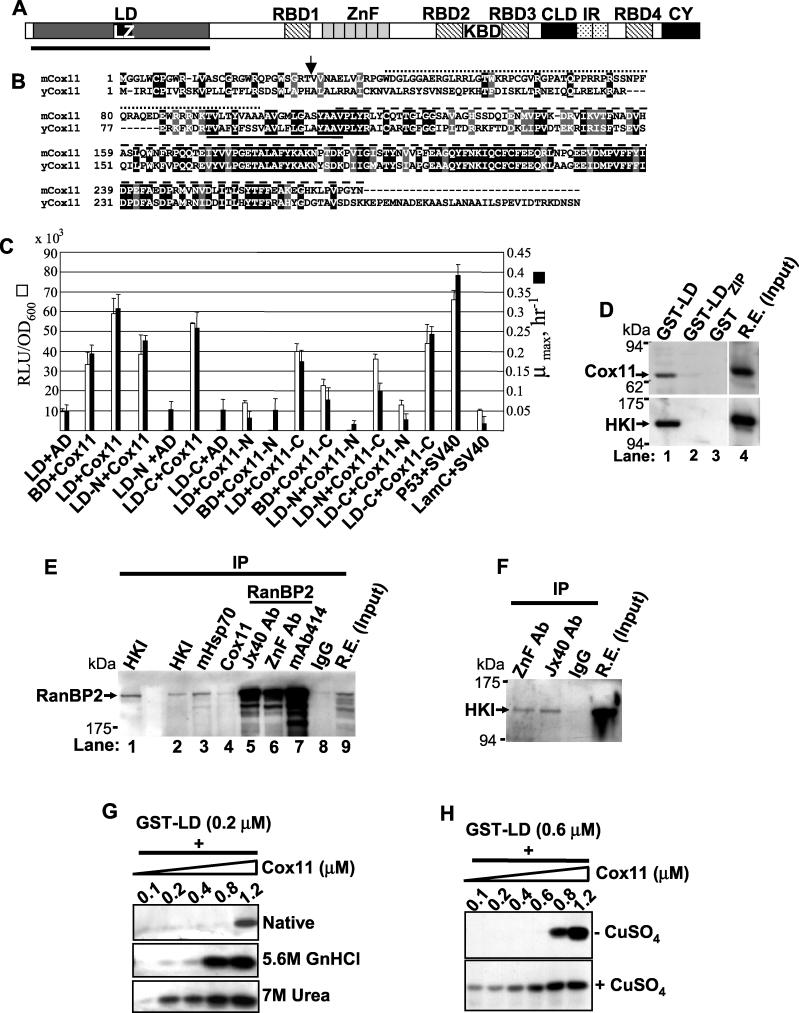
The LD of RanBP2 Interacts with Cox11 and HKI (A) Primary structure of RanBP2 and its structural/functional domains. The N-terminal LD of RanBP2 is underlined. (B) Sequence alignment of murine and yeast Cox11. The yeast Cox11 C- and N-terminal domains are poorly conserved. Arrow and solid line denote the predicted mitochondrial cleavage site and membrane-spanning domain. The dotted and dashed lines above the aligned sequences represent, respectively, Cox11-N and Cox11-C constructs shown in Figure 1C. (C) Structure-function analysis of the interaction between the LD of RanBP2 and Cox11. Optimal interaction between the LD and Cox11 occurred in the presence of constructs comprising both the complete LD and Cox11. Although removal of the cytosolic N-terminal (Cox11-C) significantly decreased the interaction with LD, the mitochondrial intermembrane domain of Cox11 (Cox11-C) together with the C-terminal half of LD (LD-C) retained most of the interaction activity. LD-N and LD-C ended and began with the leucine zipper domain of RanBP2. White and black bars denote β-galactosidase activity and growth rates in selective growth medium, respectively. Results shown represent the mean ± SD, *n* = 3. (D) GST pull-down assays with the LD of RanBP2 and its leucine zipper domain and retinal extracts. The LD, but not the leucine zipper domain of RanBP2, associate with Cox11 (top panel, lane 1) and HKI (bottom panel, lane 1). (E) Coimmunoprecipitation of RanBP2 with antibodies against its molecular partners shows that RanBP2 forms a complex in vivo with HKI (lanes 1 and 2), mHsp70 (lane 3), and Cox11 (lane 4). Lanes 5, 6, and 7 are control immunoprecipitation reactions with different antibodies against the RanBP2 domains, KBD, ZnF, and XAFXFG of nucleoporins. (F) Reciprocal coimmunoprecipitation of HKI with antibodies against RanBP2 (used and shown in (E)). (G) Reconstitution pull-down assays with purified LD and increasing concentrations of native (top panel), denatured (middle panel), and partially denatured (bottom panel) Cox11, respectively, in the absence and presence of denaturating agent, GnHCl and chaotropic agent, urea. Folding intermediates (lower panel) of Cox11 exhibit the highest binding activity toward the LD of RanBP2. (H) Similar experiments as in (G) but in the presence of native Cox11 expressed in the absence (top panel) or presence (bottom panel) of CuSO_4_. The mature isoform of the metallochaperone has an increased affinity toward the LD of RanBP2. LD, leucine-rich domain; LZ, leucine zipper domain; RBD_1–4_, Ran-binding domains 1–4; ZnF, zinc finger cluster domain; KBD, kinesin (KIF5B/KIF5C)-binding domain; CLD, cyclophilin-like domain; IR, internal repeat domain; CY, cyclophilin domain.

The LD of RanBP2 (Figure 1A) is a large and orphan domain of ~700 residues (~80 kDa), for which no molecular partners have been identified until this date. Brain and retina yeast two-hybrid libraries were screened with LD. Cox11 was identified as a partner to this domain (Figure 1B and 1C). Cox11 is a metallochaperone implicated in cytochrome c oxidase assembly [30,31]. Structure-function analysis of the interaction between mCox11, LD of RanBP2, and subdomains thereof, with quantitative yeast two-hybrid assays [32], showed optimal interaction between the intact LD and Cox11 proteins (Figure 1C). Pull-down assays of retinal extracts with glutathione *S*-transferase (GST)-LD precipitates a sodium dodecyl sulfate-resistant dimer isoform of Cox11 (Figure 1D, top panel), which does not bind to GST-LD_ZIP_ alone. In addition to Cox11, we also found that other mitochondrial components such as the outer membrane-associated protein, HKI [33] (Figure 1D, bottom panel) and mHsp70 (unpublished data), associated with LD of RanBP2. This association was highly specific toward the HKI isoform, because HKII, HKIII, and glucokinase did not interact with the LD of RanBP2 (unpublished data). The interaction of Cox11, HKI, and mHsp70 with RanBP2 occurred in vivo in retinal extracts, since antibodies against these and RanBP2 coimmunoprecipitated RanBP2 (Figure 1E) and HKI (Figure 1F), respectively, and these interactions were observed across different tissues (Figure S1). Since RanBP2 exhibits chaperone activity, we assessed whether the interaction between the LD of RanBP2 and Cox11 was direct and the chaperone activity of LD toward folding species of Cox11. Reconstitution binding assays were carried out between purified LD and Cox11, fully and partially denatured with GnHCl and urea, respectively, and native Cox11 (Figure 1G and 1H). Partial denatured Cox11 exhibits significantly higher and concentration-dependent binding affinity toward LD compared with the native and fully denatured Cox11 (Figure 1G). In addition, native Cox11 purified upon expression in the presence of CuSO_4_ (a prosthetic group tightly bound to Cox11) [31], shows significantly higher binding activity toward the LD of RanBP2, than in the absence of CuSO_4_ (Figure 1H).

### Cox 11 Inhibits HKI Activity and the LD of RanBP2 Reverses the Inhibition of Cox11 over HKI

**Figure 2:**
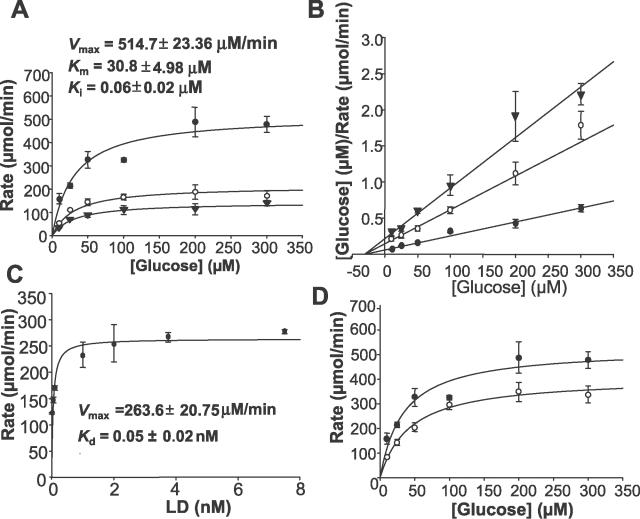
Effect of Cox11 and RanBP2 on HKI Activity (A) Saturation kinetics, rate versus glucose of HKI (0.24 μg) in the absence (solid circles) and presence of Cox11 (open circles, 0.25 nM; solid triangles, 7.5 nM). The activity of HKI decreases with increasing concentrations of Cox11. No measurable HKI activity was recorded in the presence of 15 nM of Cox11 (unpublished data). (B) Hanes-Wolf plot of (A) (1/rate versus glucose) in the absence and presence of fixed concentrations of Cox11. Linearity of reciprocal plots also supported the hyperbolic behavior of the reactions (unpublished data). Cox11 behaves as a noncompetitive inhibitor of HKI by reducing the *V*_max_ of HKI but not its *K*_m_ toward glucose. (C) HKI rate is plotted as a function of LD concentration at saturating glucose and fixed Cox11 (7.5 nM) concentrations. Note that increasing concentrations of the LD of RanBP2 reverse the inhibition of HKI activity by Cox11. A half-maximal effect of the LD of RanBP2 on HKI activity in the presence of 7.5 nM of Cox11 was observed at a concentration of ~0.05 nM of LD. (D) Rate versus glucose plot in the absence and presence of the LD of RanBP2. At a saturating concentration of the LD of RanBP2 (3.75 nM), the HKI activity was reduced by about 20%. *v,* rate; S, glucose.

To probe whether the interaction of Cox11 and HKI with the LD of RanBP2 modulates the enzymatic activity of HKI, we first examined the effect of increasing concentrations of Cox11 on the initial rates of HKI enzymatic activity (Figure 2A). Cox11 strongly inhibits HKI activity in a concentration-dependent fashion, and at ~15 nM of Cox11, HKI activity could not be recorded (Figure 2A). Cox11 behaves as a partial noncompetitive inhibitor of HKI by affecting the *V*_max_ of HKI for glucose (Figure 2B). Then, we evaluated the effect of the LD of RanBP2 on the HK activity in the presence of a fixed inhibitory concentration of Cox11, saturating concentration of glucose substrate, and increasing concentrations of LD. As shown in Figure 2C, the LD domain sharply reversed the inhibitory effect of Cox11 on HKI activity in a concentration-dependent manner, but under saturating (and stochiometric) amounts of LD, the velocity of the reaction did not reach that observed for HKI activity in the absence of Cox11 (Figure 2A), suggesting the LD by itself may also have an effect on HKI activity. Indeed, a saturating concentration of LD reduced the *V*_max_ but not the *K*_m_ of HKI (Figure 2D) by ~20% under similar conditions.

### RanBP2 Colocalizes with HK1, Cox11, and mHsp70 in the Retina and Cultured Neurons

**Figure 3:**
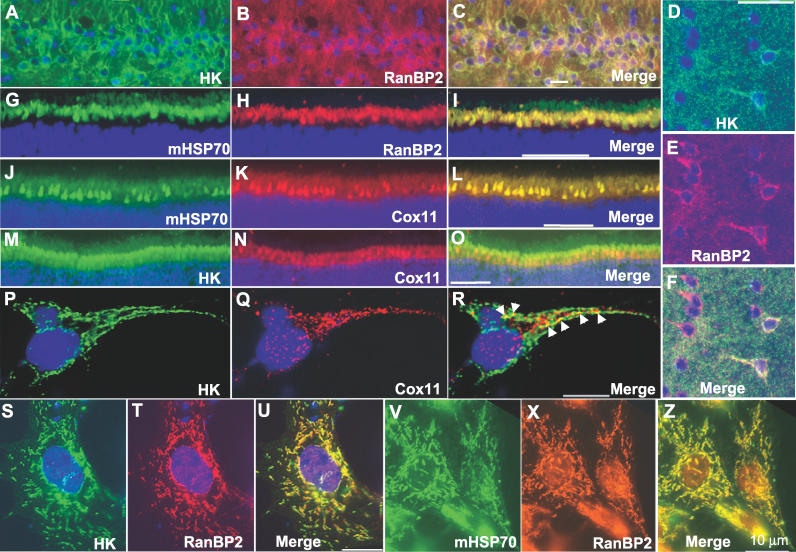
Localization of RanBP2 and Its LD Molecular Partners (A–F) are thin cryosections of an area of the hipocampus (CA1 neurons) and cerebral cortex, respectively, immunostained against HKI (A and D), RanBP2 (B and E), and merged images thereof (C and F). Note that while RanBP2 and HKI are widely expressed among and colocalize to hippocampal neurons (C), HKI expression and localization with RanBP2 is restricted to a subset of cortical neurons (likely interneurons) (F). Images of the distal region of bovine retinal cryosections comprising part of the nuclear layer of photoreceptor neurons and their inner (myoid and ellipsoid) segment compartment (G–O) are immunostained against mHsp70 (G) and RanBP2 (H), mHsp70 (J) and Cox11 (K), HKI (M) and Cox11 (N), and merged images thereof (I–O). Note the prominent localization of RanBP2, mHsp70, and Cox11 at the mitochondria-rich ellipsoid compartment of photoreceptors and the colocalization of RanBP2 and Cox11 with mHsp70 (I and L), while HKI colocalization with Cox11 was limited to restricted foci (R, arrowheads). High-resolution images of dissociated primary cerebral neurons and glial cells confirmed that the colocalization of HKI and Cox11 was highly restricted (P–R), while RanBP2 extensively colocalized with HKI (S–U) and mHsp70 (V–Z). Scale bars in A–O and P–Z are 40 and 10 μm, respectively. ONL, outer nuclear layer.

In addition to its presence at the vicinity of NPCs, we have shown previously that RanBP2 is localized to and abundant in the mitochondria-rich ellipsoid subcellular compartment of photosensory (photoreceptor) neurons of the retina [19]. We extended the subcellular colocalization studies on RanBP2 and its novel partners by immunocytochemistry to determine if RanBP2, Cox11, HKI, and mHsp70 colocalize in hippocampal neurons (Figure 3A–3C), cerebral cortex neurons (Figure 3D–3F), ellipsoid (mitochondria-rich) subcellular compartments of photosensory neurons of the retina (Figure 3G–3O), and dissociated primary glia and neuron cultures from the brain (Figure 3P–3Z). Double immunostaining with antibodies against these proteins showed that they colocalize to the mitochondria (Figure 3A–3Z).

### Haploinsufficiency of RanBP2 Causes Decreased Levels of HKI and Partial Mislocalization of HKI

**Figure 4:**
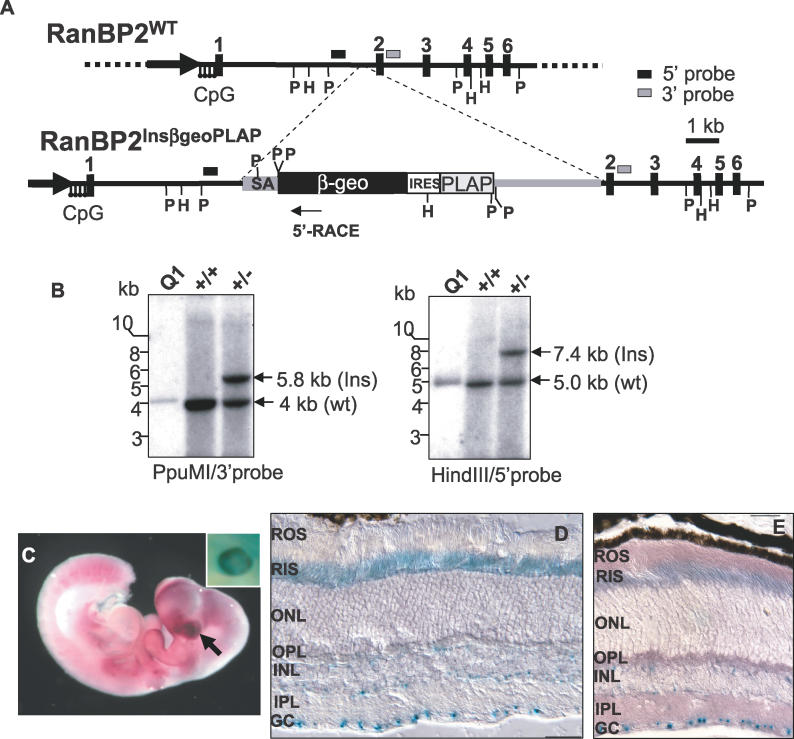
Insertion Mutagenesis of the Murine *RanBP2* Gene (A) Diagram of the genomic region of *RanBP2* disrupted by insertion trap mutagenesis with a bicistronic reporter vector between exon 1 and 2. The bicistronic transcript produces two proteins under regulation of RanBP2. Upon splicing of *RanBP2,* a fusion between exon 1 and β-geo (a fusion between the *β-gal* and *neo* genes) is generated, while human placental alkaline phophatase (PLAP) is independently translated using the internal ribosome entry site. Consistent with previous studies, the expression of the former is directed to cell bodies, while expression of the latter is targeted to the axonal processes [67,68]. Transcriptional 5′ RACE analysis detects a fusion between exon 1 and β-geo. (B) Southern analysis of the *RanBP2* locus of wild-type and heterozygous genomic DNA of tails of F1 mice digested with *Ppu*MI (left panel) and *Hind*III (right panel) with probes at the 3′ (left panel) and 5′ (right panel) flanking regions of the insertion breakpoint. Q1 is a cosmid containing the *RanBP2* gene up to exon 20 [4]. (C) Lateroventral view of a whole-mount stain of a ~12.5 dpc heterozygous embryo for PLAP and β-gal (inset picture) activities. Although PLAP was broadly expressed (e.g., somites, limbs, and CNS), the PLAP and β-Gal (inset picture) expression was particularly high in the optic vesicle (arrow). X-gal single (D) and combined staining with PLAP (E) of a retinal section of a 3-mo-old RanBP2^+/−^ mouse. Consistent with previous immunocytochemistry studies, β-Gal activity is detected in the neuroretinal bodies and inner segment compartment of photoreceptors with conspicuously strong expression in ganglion cells. PLAP expression is found throughout the plexiform/synaptic layers and outer segment of photoreceptors (E). GC, ganglion cell; PLAP, human placental alkaline phophatase; ROS, rod outer segment; RIS, rod inner segment; ONL, outer nuclear layer; OPL, outer plexiform (synaptic) layer; INL, inner nuclear layer; IPL, inner plexiform (synaptic) layer; GC, ganglion cell layer.

To determine the physiological implications of the interaction between RanBP2 and HKI (and other partners), we employed a murine embryonic stem *129Ola* cell line with a targeted *RanBP2* locus produced by gene-trapping to produce stable inbred (coisogenic) and mixed background lines, respectively (Figure 4A and 4B). Genotyping of 299 F2 offspring revealed a *RanBP2^+/+^:RanBP2^+/−^:RanBP2^−/−^* distribution (89:210:0) that deviated from the expected Mendelian ratio and supports that the *RanBP2^−/−^* mice were embryonically lethal. E12.5 embryos show strong expression of RanBP2 in the optic vesicle and throughout much of the embryo, which had no apparent developmental abnormalities (Figure 4C). In agreement with previous immunocytochemistry analysis [19], the *RanBP2* gene is expressed across mature retinal neurons, but expression in ganglion cells of the adult retina was extremely strong (Figure 4D and 4E).

**Figure 5:**
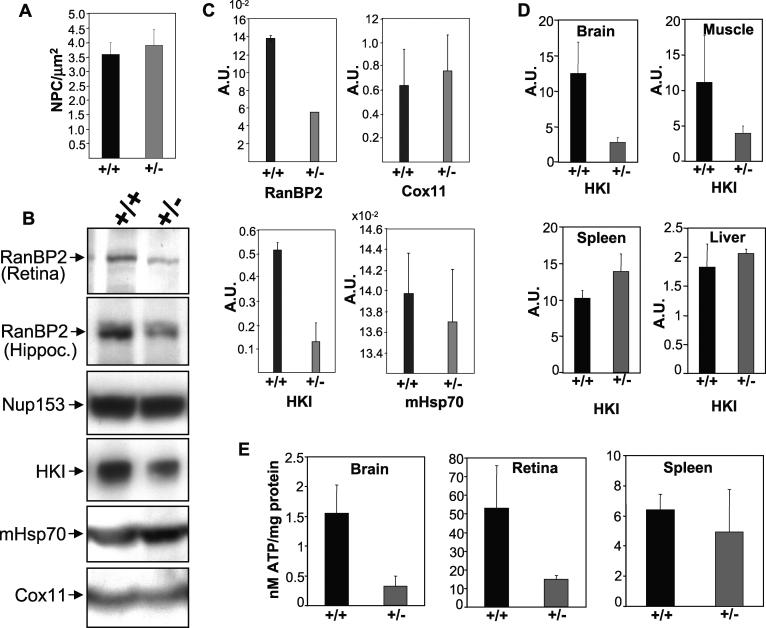
Haploinsufficiency of *RanBP2* Causes a Decrease in HKI Protein and ATP Levels (A) Quantitative analysis of NPCs in dissociated hippocampal neurons of wild-type (+/+) and heterozygote (+/−) mice upon immunostaining with mAb414. No difference in the density of NPCs (3–4 NPC/μm^2^) at the nuclear envelope was found between *RanBP2^+/+^* and *RanBP2^+/−^* mice. (B) Immunoblots with anti-RanBP2/Nup153/Nup62 (mAb414), −HKI, −mHsp70, and −Cox11 antibodies of retinal (top panel) and hippocampal homogenates of +/+ and +/− mice. In comparison to *RanBP2^+/+^, RanBP2^+/−^* mice exhibit a reduction in the expression levels of RanBP2 and HKI but not of other proteins. (C) Quantitative analysis of relative protein expression levels of RanBP2, Cox11, HKI, and mHsp70 in the hippocampus of *RanBP2^+/+^* and *RanBP2^+/−^*mice. There is ~2- and 4-fold reduction of RanBP2 and HKI in heterozygote mice. (D) The level of HKI is reduced in the brain but not in other non-neuronal tissues tested (muscle, spleen, and liver). (E) The total ATP level is reduced in the CNS tissues (brain and retina) but not in non-neuronal tissues tested (e.g., spleen).

In light of the association in vivo of RanBP2 with Cox11 and HKI (Figures 1 and 3), profound in vitro modulation of HKI enzymatic activity by RanBP2 and Cox11 (Figure 2), and the critical role of HKI in catalyzing a rate-limiting step of glycolysis, we probed whether *RanBP2^+/−^* mice presented disturbances in HKI, Cox11, and energy homeostasis. Monoallelic expression of *RanBP2* does not affect the number of NPCs and their distribution in hippocampal neurons (Figure 5A, unpublished data), but it led to consistent lower expression of RanBP2 by more than 50% in the CNS (brain and retina) in 129Ola (Figure 5B–5D), but not in C57BL/6J/129Ola backgrounds (unpublished data). Hence, we focused our analysis on the inbred *RanBP2^+/−^* 129Ola mouse line. Although the levels of RanBP2 were decreased in the retina, brain, and hippocampus of *RanBP2^+/−^*mice by ~50%–60% (Figure 5B and 5C), the levels of others nucleoporins, Nup153 and Nup62, and mHsp70 and Cox11, remained unchanged (Figure 5B, unpublished data). In addition, we observed a strong decrease of the levels of HKI (3- to 4-fold) (Figure 5B and 5C). This decrease was selective to the CNS, since HKI levels remained largely unaffected in the skeletal muscle, spleen, and liver (Figure 5D). Because HKI plays a key role in the production of energy intermediate substrates and HKI is virtually the sole HK isoform expressed in the CNS [33,34], we probed the impact of HKI and RanBP2 reduction in the levels of ATP. As shown in Figure 5E, there was significant and concordant reduction in levels of ATP in the CNS (brain and retina), but not in non-neuronal tissues. Finally, we also observed partial and selective delocalization of HKI, but not of Cox11, from the ellipsoid (mitochondria-rich) to the adjacent myoid subcellular compartment of rod photosensory neurons (Figure S2A–S2E). This was also accompanied by reduced HKI levels in the inner retina, in particular in the inner plexiform (synaptic) layer (Figure S2A–S2E).

### Metabolic Disturbances Caused by Haploinsufficiency of RanBP2

**Figure 6:**
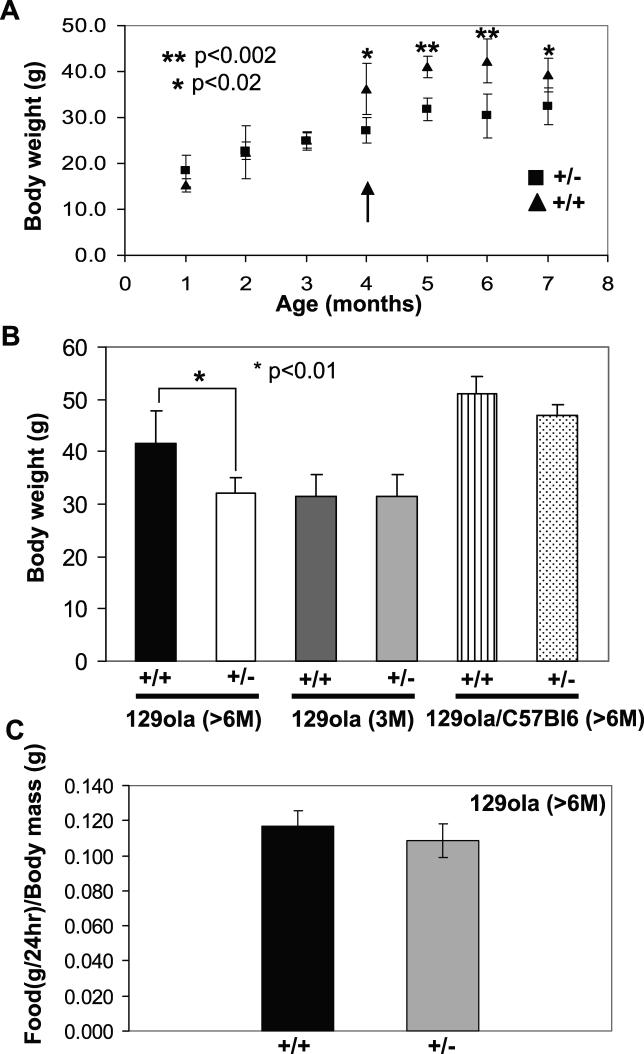
*RanBP2^+/−^* Mice on High-Fat Diet Exhibit Deficits in Growth (A) In comparison to wild-type mice, *RanBP2^+/−^* mice show slower growth rates beginning at 4 mo of age (arrow), and the difference in body weight between these is maintained afterward. Note that *RanBP2^+/−^* mice lack the growth spur observed in wild-type mice between 3 and 4 mo of age. (B) In comparison to inbred *RanBP2^+/−^*mice (129Ola genetic background), the difference in body weight between *RanBP2^+/+^* and *RanBP2^+/−^* mice is masked upon placing these on a mixed 129Ola/C57Bl6 genetic background. (C) *RanBP2^+/+^* and *RanBP2^+/−^*inbred mice exhibit similar rates of food consumption. Mice in (A), (B), and (C) were placed on a high-fat diet since birth (*n* = 5).

The growth rates of inbred *RanBP2^+/−^* mice on high-fat (~10% fat) diet were significantly slower than *RanBP2^+/+^* mice (Figure 6A). Beginning at around 4 mo of age, *RanBP2^+/−^* mice exhibit a significant slower gain in body mass than wild-type mice (Figure 6A). In addition, *RanBP2^+/−^* inbred mice presented deficits in body mass that were erased by changing the genetic background to a mixed C57BL/6J/129Ola (Figure 6B). Food consumption did not account for the body weight differences observed (Figure 6C).

**Figure 7:**
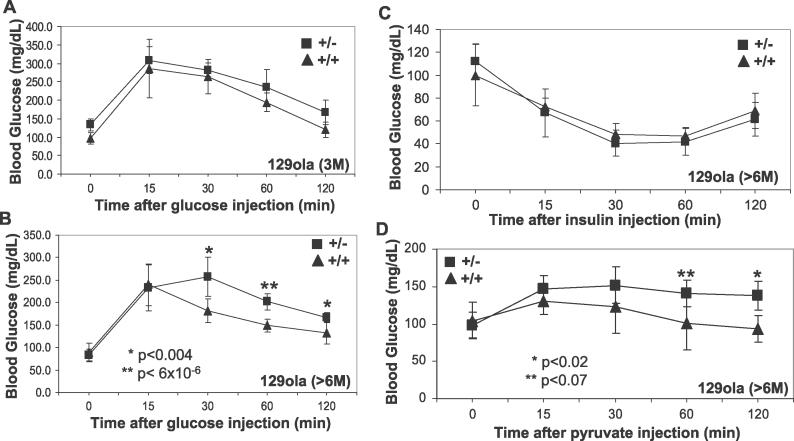
Metabolic Phenotypes of *RanBP2^+/−^* Inbred Mice on High-Fat Diet (A) 3-mo-old inbred *RanBP2^+/−^* mice (*n* = 5) have normal glucose clearance rates upon glucose challenge and overnight fasting. (B) In contrast, 6-mo-old inbred *RanBP2^+/−^* mice (*n* = 5) have significantly decreased glucose clearance rates upon glucose challenge and overnight fasting. (C) Fasted 6- to 8-mo-old *RanBP2^+/+^* and *RanBP2^+/−^* mice have no difference in insulin-mediated glucose uptake as assayed by insulin tolerance test (*n* = 5). (D) Pyruvate tolerance test shows normal rise in glucose but decreased glucose clearance between inbred *RanBP2^+/+^* and *RanBP2^+/−^* mice (*n* = 5).

HKI in the CNS (brain and retina) accounts virtually for all expression of HK isozymes and glucose utilization in the CNS [33,34]. Moreover, glucose is the sole reliance source of energy in the CNS under normal conditions, the CNS lacks glucose storage sources, and despite the disproportionate mass of the CNS to the rest of the body, the CNS consumes daily about 60% of the body's glucose and 25% of the total oxygen [35,36]. To determine the impact of *RanBP2* haploinsufficiency on the utilization, formation, and uptake of glucose, we carried out several physiological assays. In contrast to mice placed on a normal chow diet (~5% fat; unpublished data), *RanBP2^+/−^*mice on a higher fat diet (~10% fat) performed significantly worse in the glucose tolerance test beginning at 6 mo of age (Figure 7A and 7B), thus supporting that the *RanBP2^+/−^* mice exhibited a deficit in glucose clearance. This deficit was rescued in *RanBP2^+/−^* mice of mixed C57BL/6J/129Ola background (Figure S3). Glucose clearance was not affected due to a disturbance in insulin-mediated glucose uptake (Figure 7C). Then, we probed whether RanBP2 induces impairment of gluconeogenesis, which could contribute to the pathophysiological production and clearance of glucose. To this end, the administration of the gluconeogenic substrate precursor, pyruvate (pyruvate tolerance test), showed that there was no difference in glucose production in *RanBP2^+/−^* mice (Figure 7D). Hence, partial loss-of-function of *RanBP2* had no impact on the gluconeogenesis pathway. However, upon glucose production (15 min), the clearance rates of glucose were again significantly slower in *RanBP2^+/−^* than in *RanBP2^+/+^* mice (Figure 7D), confirming an impairment in glucose breakdown.

### Haploinsufficiency of RanBP2 Causes Deficits in the Electrophysiological Output of Receptoral and Postreceptoral Retinal Neurons

**Figure 8:**
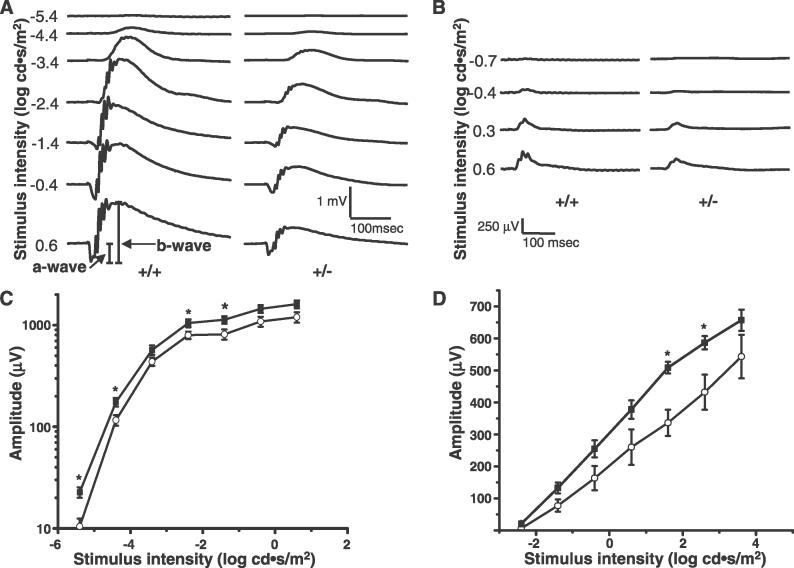
Electroretinograms from 6-Mo-Old *RanBP2^+/−^*and *RanBP2^+/+^* Inbred Mice Showing Photoreceptor and Postreceptor Neuron Electrophysiological Response Phenotypes (A) Scotopic (dark-adapted) responses from *RanBP2^+/−^* mice to light stimuli of increasing intensity, beginning at threshold, have reduced amplitudes compared to those observed in *RanBP2^+/+^* mice. The three lower intensities represent responses generated in the rod photoreceptor neuronal pathway. The upper intensities are comprised of responses generated in both the rod and cone pathways. (B) Photopic (light-adapted, cone photoreceptor pathway) responses of *RanBP2^+/−^* mice to increasing light stimulus intensities also exhibited reduced amplitudes compared to those observed in *RanBP2^+/+^* mice. (C) Average ± SE (*n* = 9) scotopic *b*-wave amplitudes from *RanBP2^+/−^* (open circles) and *RanBP2^+/+^* (filled squares) mice representing postreceptoral neuron function. (Note: log amplitude scale.) (D) Average ± SE (*n* = 5) scotopic *a*-wave amplitudes, representing photoreceptor function, for *RanBP2^+/−^* and *RanBP2^+/+^* mice in response to bright flashes. Amplitudes of responses from *RanBP2^+/−^* mice were lower over the entire range of stimulus intensities for both *b*- and *a*-waves. Asterisks represent significant differences between the groups (Student's *t* test, *p* < 0.05). Statistical significance was found across all intensities for *b*-wave amplitudes (2-way ANOVA, *p* < 0.0001), but not for the *a*-wave.

In light of the prominent expression of RanBP2 and HKI in retinal neurons [1,19], the vital dependence of the neuronal retina (and brain) on glucose as the main substrate source for energy production, and the determinant impact of metabolic disorders, such as diabetes, in retinal function (e.g., diabetic retinopathy) [37], we probed the impact of deficits in RanBP2, HKI, and ATP, on the electrophysiological responses of subclasses (rod and cone) photoreceptor and postreceptor retinal neurons of *RanBP2^+/−^* and in *RanBP2^+/+^* mice. The scotopic (dark-adapted) responses mediated by the rod photoreceptor pathway at low-stimulus intensities and mixed rod and cone pathways at high-stimulus intensities were substantially reduced in *RanBP2^+/−^* mice (Figure 8A). The differences in the photopic (light-adapted) responses, initiated by cone photoreceptors, which make up 3% of the photosensory neurons in the mouse retina [38], were less obvious but still exhibited a trend toward reduced amplitudes across a range of increasing light stimulus intensities (Figure 8B). The reduction in the scotopic responses included decreases in both *b*-wave (Figure 8C) and *a*-wave (Figure 8D) amplitudes mediated by postreceptoral and receptoral neurons, respectively. Postreceptoral second-order neuron responses, represented by the *b*-wave, tended to be more consistently and substantially reduced than the *a*-waves, which directly reflect photoreceptor activity. Since second-order neuron responses depend on input from photoreceptors, this suggests that reduced *b*-wave amplitudes are the result of the accumulation of decreases in the light response of both photoreceptors and postreceptoral neurons. Anesthetics, particularly ketamine, can cause sustained elevation of glucose in mice, which in turn affects electroretinogram responses [39]. Thus, we were concerned that differences in electroretinogram amplitudes between *RanBP2^+/−^* and *RanBP2^+/+^* may reflect differences in glucose level changes in response to anesthesia. However, we found no significant differences in glucose levels measured before and every 15 min during 75 min of anesthesia (*n* = 4–5). Glucose rose at the same rate and reached a maximum of approximately 3.3 times the pre-anesthesia level in both genotypes (unpublished data).

## Discussion

Our findings support that RanBP2 plays a determinant role in modulating glucose and energy homeostasis. The data support that Cox1 and HKI are novel partners in vivo for the large LD of RanBP2 (Figures 1 and 2). The LD of RanBP2 exhibits chaperone activity toward folding intermediates of Cox11, and possibly, the mature HKI (Figure 1). Cox11 inhibits noncompetitively the activity of HKI with ~2–3 molecules of Cox11 (assuming formation of Cox11 dimer) required to inhibit completely the activity of a molecule of HKI (Figure 2A and 2B). Cox11 sequesters HKI by binding to HKI at a site that is distinct from the active site and effectively reduces the availability of [HKI]_tot_ for catalysis. This is reflected by a reduction of the *V*_max_ of HKI without significantly affecting the *K*_m_ of the active site of HKI toward glucose (Figure 2A and 2B). The inhibitory property of Cox11 over HKI is suppressed by RanBP2 (Figure 2C), which by itself has a weak but significant inhibitory activity over HKI (Figure 2D). The sub-stochiometry effect of the LD of RanBP2 over the inhibition of HKI by Cox11 supports that a LD-dependent chaperonin-like mechanism underlies the suppression of Cox11-dependent inhibition of HKI by RanBP2, and that RanBP2 acts as a molecular “buffer” over HK1 and Cox11 activities. The partial loss of the RanBP2 chaperone activity in *RanBP2^+/−^* mice also leads to deficits in the sequestration of HKI in the ellipsoid compartment of photosensory neurons. This possibly underlies the mistargeting of HKI to the myoid compartment of these neurons (Figure S2). The data support that the ultimate pathophysiological outcome in HKI, caused by a reduction in RanBP2 levels and its chaperone activity, is the selective degradation of HKI as reflected by the reduced levels of HKI (and ATP) but not of other mitochondrial and NPC components (Figure 5). Through this process, it is also possible that deficits in RanBP2 cause a disturbance in the equilibrium between Cox11, HK1, and RanBP2 by leading to an increase of the inhibitory activity of Cox11 over HKI that promotes the uncoupling of the interaction of HKI from RanBP2, ultimately causing HKI degradation. Regardless, lower levels of HKI likely contribute to the decreased levels in ATP, slower growth rates, and diminished ability to metabolize glucose of *RanBP2^+/−^* mice (Figures 5–7). As discussed subsequently, the ATP deficits in the retina likely account for the reduced electrophysiological responses of retinal neurons (Figure 8).

Various domains of RanBP2 have been previously implicated with a chaperone role in the cell. These include the enhancement of the biogenesis of red/green opsin by the combination of the RBD4-CY domains [6,7] and the stabilization by the Ran-binding domains of RanBP2 of the guanosine triphosphate-bound conformational state of RanGTPase and interaction of Ran with importin-β [11,40,41]. The data herein show that the multi-chaperone role of RanBP2 extends also to its LD in light of its ability to associate to distinct folding species of Cox11 and to prevent HKI from being degraded. This chaperone function is likely to be complemented by other partners of RanBP2 with similar and pleiotropic functions. For example, the combination of the CLD of RanBP2 with several subunits of the 19S cap of the proteasome [14], and of its neighboring internal repeat, W1W2/IR with the E3-ubiquitin ligase, parkin [20], and the E2 SUMO-1-conjugating protein, Ubc9 [15] may contribute to the down-regulation of HKI by 26S proteasome-mediated proteolysis and modulation of the molecular and subcellular partitioning of these partners. In this regard, it will be interesting to probe whether parkin also causes deficits in HKI in dopaminergic neurons, since parkin was reported to modulate RanBP2 turnover [20], and parkin loss-of-function also causes energy and growth deficits [26–29,42]. These data add a new dimension to the complexity of the regulation of the glycolytic pathway, in particular in the CNS, where glycolysis plays a major role in supplying energy and where the proteasome machinery may play a critical role in modulating components of the energy supply machinery [43]. This is further evidenced by the presence of genetic modifiers in *RanBP2^+/−^*mice on a mixed genetic background that compensates for deficits in RanBP2 and deregulation of its partners in glucose/energy homeostasis as observed in the coisogenic line. The presence of such compensatory mechanisms is also supported by the identification of a quantitative trait locus, which encompasses *RanBP2,* and modifies the expression of diabetes-related phenotypes [44].

**Figure 9:**
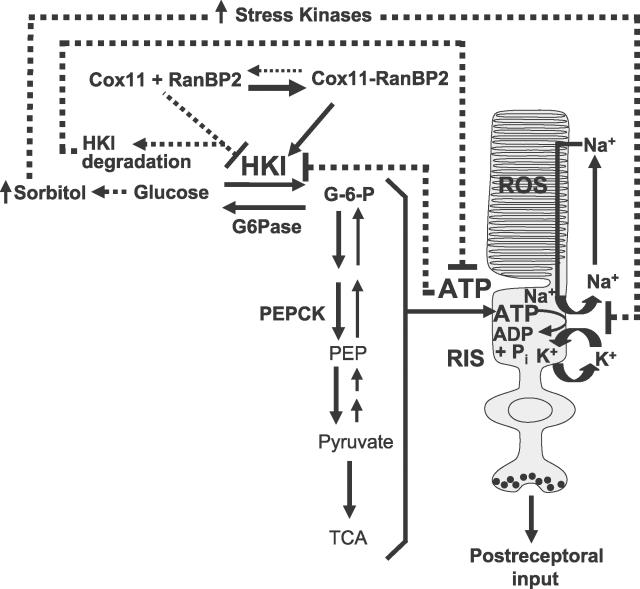
Model Depicting a Role of RanBP2 and Some of Its Partners in Metabolic and Neuronal Function RanBP2 interacts with Cox11 and HKI and the triad is in equilibrium under normal physiological conditions. RanBP2 prevents the inhibition of HKI by Cox11 and its degradation. The ultimate effect of RanBP2 on its partners is the stimulation of the glycolytic pathway and production of ATP. The glycolytic pathway is critical to fuel the constitutive Na^+^/K^+^-ATPase pump to maintain the dark current between the inner and outer segment compartments of photosensory neurons. A deficit (haploinsufficiency) in RanBP2 disturbs the equilibrium between RanBP2, HKI, and Cox11. This pathophysiological event promotes the destabilization and degradation of HKI and a decrease in ATP production required to maintain the depolarization state neurons, and, hence, a reduction in the response of receptoral and postreceptoral neurons. A reduction in ATP levels also negatively modulates HKI activity/level. Decreased levels of HKI promote intracellular hyperglycemia and activate stress kinases, which modulate negatively the Na^+^/K^+^-ATPase pump by phosphorylation. Pathophysiological pathways promoted by RanBP2 haploinsufficiency are represented by dash lines. RIS, rod inner segment; ROS, rod outer segment.

The data herein show tissues with a decrease of RanBP2 and HKI levels mirror a reduction in the ATP levels. Since the constitutive Na^+^/K^+^ ATPase pump and the ATP-dependent conversion of glutamine to glutamate (both fundamental to maintain the electrical activity in the CNS) consumes the vast majority of the energy produced by the CNS [45–47], this likely underlies the suppression of the electrophysiological output responses of retinal neurons (Figure 8). Moreover, deficits in HKI may lead to intracellular hyperglycemia in the CNS, promote sorbitol-induced osmotic stress, and compromise further the ATPase-dependent Na^+^/K^+^ pump activity [48–50]. This may be exacerbated by a decrease of ATP, since the activity of HKI is also stimulated by ATP [51]. The cumulative effects of a reduction in ATP and intracellular hyperglycemia are known to act synergistically and modulate the electrophysiological properties of neuronal activity. Figure 9 integrates in a model these variables and implications of the data presented herein. Still, other bona fide RanBP2 partners previously identified and described in the Introduction also become strong candidates to play a role in energy homeostasis. For example, the unfolding and chaperone activity of components of the 19S cap of the proteasome may be modulated by the CLD of RanBP2 [14] and contribute to the selective HKI (and other substrates) degradation [43], while association of nuclear import receptor, importin-β with the Ran-binding domains of RanBP2 [10,11], may mediate the nuclear translocation of multifunctional substrates, such as glyceraldehyde-3-phosphate dehydrogenase. This trafficking process is selectively inhibited by the neuroprotective drug, *R-*(-)-deprenyl and derivates thereof, and are often employed to treat Parkinson disease [52–57]. Hence, RanBP2 and its partners emerge as key players and target genes in mediating neuropathophysiological mechanisms implicated in various genetic and environmental lesions to the CNS, as in patients with Parkinson, diabetes with insulin-resistance, and other neuropathies and neurodegenerative diseases, often linked to aging manifestations. To this effect, the RanBP2 mouse model will serve as a unique genetic tool to probe selective, multiple and novel pathways, which may have not been anticipated to be linked to metabolic processes and allied pathophysiological states.

Finally, RanBP2 appears to join other nucleoporins, such as Nup96 [58], in novel physiological functions that are vital for complex organisms and that were not anticipated from cell-based studies. The reduced expression of these proteins in whole-animal models generates phenotypes that are tissue-restricted and more importantly, do not seem to recapitulate phenotypes observed with knockdown experiments in cell-based culture assays. For example, short interfering RNA-mediated knockdown of RanBP2 [9] and members of the Nup107-Nup160 complex [59–61] have shown, respectively, to cause mitotic arrest and disruption of nuclear pore assembly and deficits in mRNA export in cell culture. Yet, haploinsufficiency of RanBP2 (this work) and Nup96 [58] predominantly produce, instead, CNS-restricted deficits in energy metabolism and alterations in the immune system linked to the down-regulation of interferon-β-regulated proteins and increased susceptibility of viral infection, respectively. While these apparent outcome disparities among experimental systems remain unclear, they could potentially result from variations in redundancies and compensatory mechanisms inherent to each experimental system. Regardless, these genetic and whole-animal models set the stage to probe novel molecular pathways in various physiological and genetic contexts, and provide also a link to pathophysiological processes underlying several human diseases.

## Materials and Methods

### Yeast two-hybrid screening and assays.

The LD of human RanBP2 (residues 62–711) was subcloned in-frame into the GAL4 DNA-binding domain of bait vector, pBUTE (a kanamycin-resistant version of GAL4 bait vector pGBDUC1) [62] and HybriZAP pBD-GAL4 vector (Stratagene, La Jolla, California, United States). The former was used to screen ~18 million clones via mating from murine 9- to 10-d-old embryo and brain cDNA libraries at the Molecular Interaction Facility (MIF), University of Wisconsin, Madison, Wisconsin. The latter was used to screen via transformation of ~5 million clones from bovine retinal cDNA libraries [32]. The screens generated six clones. One and two in-frame clones were independently isolated from the embryonic and adult brain libraries, respectively, and the interactions were validated. The three clones encoded Cox11. Interactions between Cox11, LD, and subdomains thereof were quantified by liquid β-galactosidase (Applied Biosystems, Foster City, California, United States) and growth assays [63]. The maximum specific growth speed (μ_max_) was determined by calculating μ_max_ = (ln(*x*_t_) − ln(*x*_0_))/t, where *x*_t_ is the OD_600_ of the culture at t = t, *x*_0_ is the OD_600_ at t = 0 and t is the time between *x*_0_ and *x*_t_. Assays were performed with three independent clones and three samples of each clone, the results were averaged, and the standard deviations calculated.

### Site-directed and deletion mutagenesis and plasmid construction.

Deletion mutagenesis was carried out with pairs of primers against domains of interest described in the figure legends. PCR products were subcloned into pGEX-KG [64], HybriZAP pBD-GAL4 vector (Stratagene), and pBUTE [62] vectors. GST-LD_ZIP_ alone comprised residues 447–483 of human RanBP2.

### GST pull-down and immunoprecipitation assays.

CHAPS-solubilized retinal extracts, expression, and purification of GST-fused constructs were prepared as previously described [65]. GST pull-down assays were carried out with 0.5–2.2 μM of GST-fused proteins [65]. Co-precipitates were resolved on SDS-PAGE and analyzed by Western blot with antibodies described in the Results section. Unfolded and partially denatured Cox11 were generated by incubating recombinant and native Cox11 overnight with 5.6 and 7 M guanidine hydrochloride and urea, respectively. The Cox11 conformers were then diluted ~20-fold in CHAPS-binding buffer containing GST-LD. Immunoprecipitation assays with 5 μg of antibody and Western blots (~ 200–400 ng/ml of antibody) were performed exactly as described previously [13].

### Hexokinase I assay.

Hexokinase I activity was determined spectrophotometrically at 25 °C by the method of coupling the glucose-6-phosphate production via glucose-6-phosphate dehydrogenase with the change in the absorbance of NADPH at 340 nm and as described by [66]. The reaction was started by the addition of purified brain hexokinase I (0.24 μg) (gift from J. Wilson) to 1.0 ml of reaction mixture containing 0.05 M Tris-HCl (pH 8.5), 7.4 mM MgCl_2_, 6.6 mM ATP, 0.65 mM NADP, 11.1 mM monothioglycerol, and 1 unit of glucose 6-phosphate dehydrogenase. In the case of hexokinase activity measured in the presence of Cox11 and LD, the purified HKI was incubated with the recombinant proteins for 15 min at 4 °C before measurement of the activity. The data were fitted directly into the Michaelis-Menten equation using SIGMAPLOT (SPSS Science).

### Antibodies.

Rabbit antisera were raised against the recombinant murine Cox11 (residues 40–275) and affinity-purified under non-denaturing conditions (Stereogene) as previously described [13]. Cox11 antibodies were used at 200 ng/ml for Western analysis. The monoclonal antibody Hsp70 was from Affinity Bioreagents (Golden, Colorado, United States) and antibodies against the KBD (JX2) and ZnF domains of RanBP2 have been described [13,16]. Polyclonal and monoclonal antibodies against Hexokinase I were provided by J. Wilson. The mAb414 was purchased from Abcam (Cambridge, Massachusetts, United States).

### Immunocytochemistry and microscopy.

Retina dissections, radial cryosections (~6 μm), and immunohistochemistry procedures were carried out as described elsewhere [19]. Brains from 3- to 5-mo-old C57Bl/6 mice were fixed overnight in 2% paraformaldehyde, infused with 30% sucrose, and processed for cryosectioning. Primary brain neurons and glial cells were prepared from the cerebral cortex. This was macerated in the Hank's Balanced Salt solution and triturated. Primary cells were cultured in DMEM (GIBCO, San Diego, California, United States) and collagen-coated 35-mm glass bottom culture dishes (MatTek Corporation, Ashland, Maine) at 5% CO_2_/37 °C for ~2 d and processed for immunocytochemistry. Primary antibodies were used at concentrations ~2.5–10 μg/ml. Alexa 488- and Alexa 594-conjugated secondary antibodies (2.5 μg/ml) (Molecular Probes) were used for visualization of proteins. Crossover of fluorescent probes, background, and autofluorescence were found to be negligible. Visualization of specimens and localization of proteins were carried out by wide-field epifluorescence microscopy on Nikon (Tokyo, Japan) E600 upright and TE2000U inverted research microscopes equipped with similar Apochromat objectives. Images with the Nikon E600 were acquired with a SPOT-RT digital camera coupled to the microscope and driven by SPOT Imaging v4.0 software (Diagnostic Instruments, Sterling Heights, Michigan, United States). All images were captured at nonsaturating integration levels, 12-bit mono black/white, and then pseudo-colored. Protein colocalization analysis was carried out on the Nikon TE2000U microscope equipped with appropriate excitation and emission filter wheels, cube filters, 100 W mercury light source, Nomarski/DIC, and Plan Apochromat optics (100×, 60×, and 40× oil objectives with NA of 1.4, 40 ×LWD, and 20 ×LWD objectives and encoded motorized Z-Stage (Prior Scientific, Rockland, Maryland, United States). Images with the inverted Nikon TE2000U microscope were obtained using a CCD camera (CoolSNAP HQ; Roper Scientific, Trenton, New Jersey, United States). Images acquired under identical acquisition parameters were analyzed using Metamorph Software v6.2 (Universal Imaging, Glendale, Wisconsin, United States). Whenever applicable, serial optical Z-stacks (20–30 focal planes at 100-nm intervals) were captured and computationally processed by 3-D blind deconvolution methods with the same software.

### Generation of a mouse line with a disrupted RanBP2 locus and histological analysis of trapped RanBP2 mice.

A feeder-independent mouse ES cell line derived from the 129P2/OlaHsd strain and harboring a disrupted *RanBP2* locus by insertion mutagenesis with the gene trap vector, pGT0pfs [67,68] (generated by W. Skarnes), was obtained from the Mutant Mouse Regional Resource Center (University of California Davis, Davis, California, United States). The gene trap vector contains a promoterless neo-*lacZ* fusion gene with a splice acceptor site at the 5′ end followed by an internal ribosome entry site (IRES) and the human placental alkaline phosphatase (PLAP) reporter cassette. The *RanBP2* 129Ola ES line was injected into C57BL/6J blastocysts and four male chimeras were generated. These were bred into 129P2/OlaHsd (Harlan, Indianapolis, Indiana, United States) and C57BL/6J (Jackson Laboratory, Bar Harbor, Maine, United States) females. The two resulting and independent F1s lines, with 129P2/OlaHsd inbred and 129P2/OlaHsd/C57BL/6J mixed genetic backgrounds, were tested for germline transmission by PCR and Southern blot analysis of tail genomic DNA. β-gal and PLAP activities in whole mount embryos and retinal sections were detected, respectively, by incubation with 5-bromo-4-chloro-3-indolyl β-D-galactopyranoside and AP staining buffer (0.1 mg/ml 5-bromo-4-chloro-3-indolyl phosphate, 1 mg/ml nitroblue tetrazolium in 100 mM Tris-HCl (pH 9.5), 100 mM NaCl, 5 mM MgCl_2_) as described elsewhere [67,68].

### Western blot and quantitation analysis of protein expression and ATP levels.

Homogenates and detergent solubilized extracts of tissues and immunoblots were prepared as described elsewhere [69]. Quantitation of immunoblot bands calibrated against internal marker bands, such as mHsp70 and Nup153, was carried out with Gel Pro Analyzer (MediaCybernetics, San Diego, California, United States). For determination of ATP levels, freshly dissected and flash-frozen tissues were homogenized in 2.5% trichloroacetic acid (TCA), neutralized, and diluted to a final concentration of 0.1% with Tris-Acetate (pH 7.75). ATP measurements were carried out with the ENLITEN ATP Assay System Bioluminescence Detection Kit for ATP (Promega, Madison, Wisconsin, United States) as per the manufacturer's instruction in SpectraMax-M5 (Molecular Devices, Sunnyvale, California, United States). Protein concentration was measured by the BCA method (BioRad, Hercules, California, United States) using BSA as a standard.

### Metabolic assays.

Two groups of male mice were kept under separate diet since gestation or birth. One group was fed with a normal chow diet (PicoLab Rodent Diet 20 −5053; LabDiet, Richmond, Indiana, United States), while the other was placed on a higher energy and fat diet (PicoLab Mouse Diet 20 −5058; LabDiet). Glucose tolerance test was performed on overnight-fasted mice injected intraperitoneally with 2 g of glucose per kg of body weight. Venous blood glucose values were determined at immediately before (0 min), and 15, 30, 60, and 120 min after the injection with an automatic glucose monitor instrument (One Touch Ultra, Lifescan). Insulin tolerance test was performed the same way as that described for the glucose tolerance test with the exception that human insulin (0.75 U/kg of body mass) was injected intraperitoneally in non-fasted mice. Pyruvate challenge test was performed the same way as that described for the glucose tolerance test with the exception that pyruvate dissolved in saline (2 g/kg of body mass) was injected intraperitoneally in overnight-fasted mice.

### Electroretinography.

Five ~6-mo-old male *RanBP2^+/+^* and *RanBP2^+/−^* mice in *129ola* background were kept in dim cyclic light (10 lux) for 2 wk before electroretinographic responses were recorded. The mice were anesthetized intraperitoneally with ketamine (80 mg/kg) and xylazine (4 mg/kg). Body temperature was maintained with a heating pad at 37.5 ± 1 °C throughout the recording. The pupils were fully dilated with 0.5% tropicamide and 2.5% phenylephrine HCl, and both eyes were recorded simultaneously. Corneal gold-wire loops were used as the active electrodes under 0.5% proparacaine hydrochloride topical anesthesia. Gold-wire electrodes placed on the sclera near the limbus served as reference electrodes. A ground wire was attached to the ear. Scotopic responses were recorded from threshold to 9 log units of intensity above threshold, amplified, and filtered (5,000 gain, 0.1–1,000 Hz). For photopic recordings background light at 34 cd/m^2^ was used to suppress rod function.

## Supporting Information

Figure S1 Western Blot Analysis of the Association of LD of RanBP2 with HKI and Cox11 across Tissues GST-JX2 (2.2 μM) was incubated with different tissue extracts (~2.0 mg) and the GST-JX2 coprecipitates from seven different tissues were analyzed by Western blot with the antibodies against HKI and Cox11. The LD of RanBP2 associated with the HKI and Cox11 in all seven tissues tested.
(59 KB PDF)

Figure S2 Immunostaining of Radial Cryosections of Wild-Type (+/+) and Heterozygote (+/−) *RanBP2* Mice Retinas with Anti-HKI and −Cox11 Antibodies A1 and B1 represent magnifications of just the distal section of the retina (inner segment subcellular compartment of the photosensory neuron layer). Note the partial delocalization of HKI to the myoid subcompartment of the inner segment of photoreceptor neurons in +/− mice (B1) and decreased levels of HKI in the inner plexiform (synaptic) layer of the retina (A and B). This is reflected by a significant increase and decrease of the mean fluorescence intensity (in arbitrary units) of HKI in the myoid and inner plexiform (synaptic) layers, respectively (E). AU, arbitrary unit; ROS, rod outer segment; RIS, rod inner segment; ONL, outer nuclear layer; OPL, outer plexiform layer, INL, inner nuclear layer; IPL, inner plexiform layer; GCL, ganglion cell layer; E and M are, respectively, the ellipsoid (mitochondria-rich) and myoid compartments of photosensory neurons.
(476 KB PDF)

Figure S3 Metabolic Phenotype of *RanBP2^+/−^* Mice in a Mixed Background and High-Fat Diet There was no difference in the glucose tolerance test between *RanBP2^+/+^* and *RanBP2^+/−^*mice on a mixed 129Ola/C57Bl6 genetic background (C).
(38 KB PDF)

## Abbreviations

CLD: cyclophilin-like domain
CNS: central nervous system
GST: glutathione-*S*-transferase
HKI: hexokinase type I
LD: leucine-rich domain
NPC: nuclear pore complex
RanBP2: Ran-binding protein 2
