# Supplementary figures and images for: RanBP2 Modulates Cox11 and Hexokinase I Activities and Haploinsufficiency of RanBP2 Causes Deficits in Glucose Metabolism
Source: PLoS Genet. 2006 Oct 27;2(10):e177. doi: 10.1371/journal.pgen.0020177 (PMC1626108; doi:10.1371/journal.pgen.0020177)

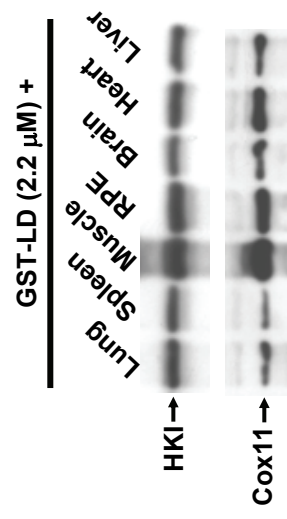

Figure S1

Supplement: Figure S1 — GST-JX2 (2.2 μM) was incubated with different tissue extracts (~2.0 mg) and the GST-JX2 coprecipitates from seven different tissues were analyzed by Western blot with the antibodies against HKI and Cox11. The LD of RanBP2 associated with the HKI and Cox11 in all seven tissues tested. (59 KB PDF) [file pgen.0020177.sg001.pdf]

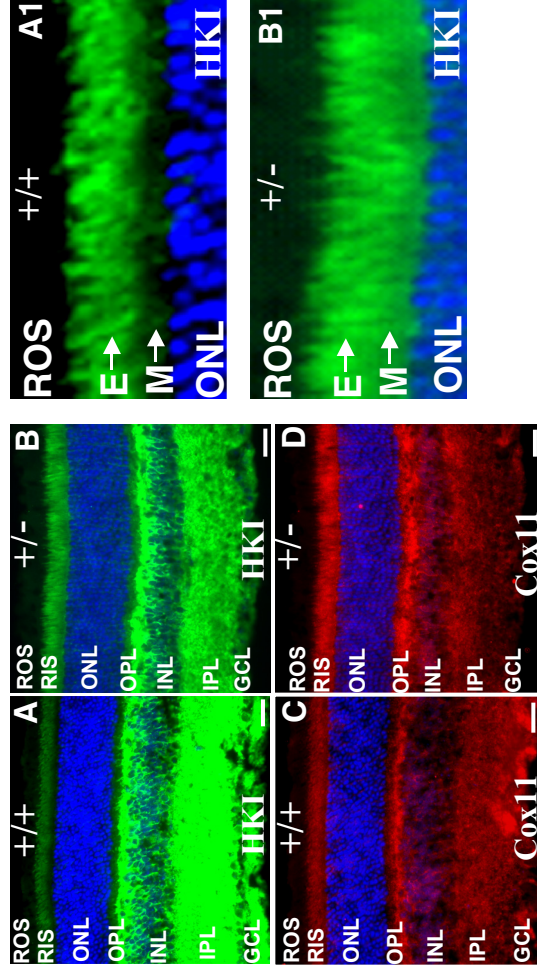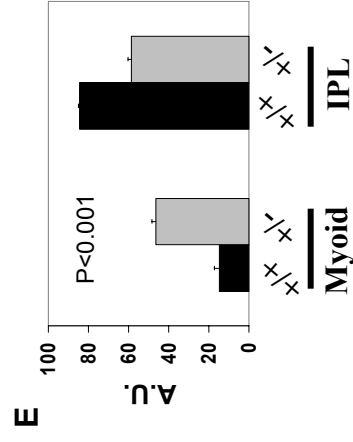

## Figure S2

Supplement: Figure S2 — A1 and B1 represent magnifications of just the distal section of the retina (inner segment subcellular compartment of the photosensory neuron layer). Note the partial delocalization of HKI to the myoid subcompartment of the inner segment of photoreceptor neurons in +/− mice (B1) and decreased levels of HKI in the inner plexiform (synaptic) layer of the retina (A and B). This is reflected by a significant increase and decrease of the mean fluorescence intensity (in arbitrary units) of HKI in the myoid and inner plexiform (synaptic) layers, respectively (E). AU, arbitrary unit; ROS, rod outer segment; RIS, rod inner segment; ONL, outer nuclear layer; OPL, outer plexiform layer, INL, inner nuclear layer; IPL, inner plexiform layer; GCL, ganglion cell layer; E and M are, respectively, the ellipsoid (mitochondria-rich) and myoid compartments of photosensory neurons. (476 KB PDF) [file pgen.0020177.sg002.pdf]

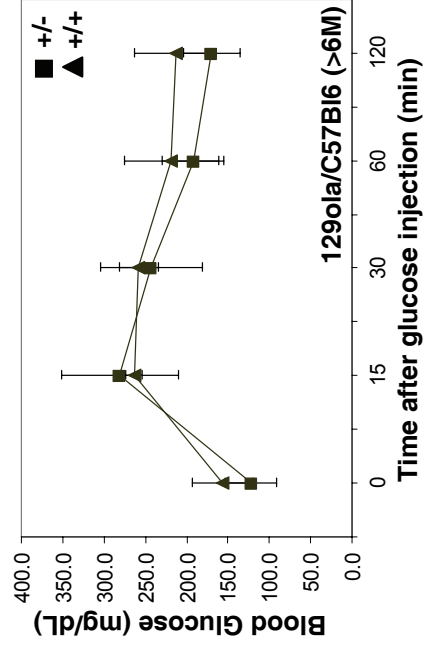

Figure S3

Supplement: Figure S3 — There was no difference in the glucose tolerance test between RanBP2+/+ and RanBP2+/−mice on a mixed 129Ola/C57Bl6 genetic background (C). (38 KB PDF) [file pgen.0020177.sg003.pdf]
